# Interpretable Severity Scoring of Pelvic Trauma Through Automated Fracture Detection and Bayesian Inference

**DOI:** 10.1109/TMI.2024.3428836

**Published:** 2025-01-02

**Authors:** Haomin Chen, David Dreizin, Catalina Gomez, Anna Zapaishchykova, Mathias Unberath

**Affiliations:** Department of Computer Science, Johns Hopkins University, Baltimore, MD 21218 USA; Department of Diagnostic Radiology, University of Maryland at Baltimore, Baltimore, MD 21201 USA; Department of Computer Science, Johns Hopkins University, Baltimore, MD 21218 USA; Department of Computer Science, Johns Hopkins University, Baltimore, MD 21218 USA. She is now with AIM Lab - Harvard MGB, Boston, MA 02115 USA; Department of Computer Science, Johns Hopkins University, Baltimore, MD 21218 USA

**Keywords:** Bayesian inference, deep learning, explainable machine learning, human-computer interaction

## Abstract

Pelvic ring disruptions result from blunt injury mechanisms and are potentially lethal mainly due to associated injuries and massive pelvic hemorrhage. The severity of pelvic fractures in trauma victims is frequently assessed by grading the fracture according to the Tile AO/OTA classification in whole-body Computed Tomography (CT) scans. Due to the high volume of whole-body CT scans generated in trauma centers, the overall information content of a single whole-body CT scan and low manual CT reading speed, an automatic approach to Tile classification would provide substantial value, e. g., to prioritize the reading sequence of the trauma radiologists or enable them to focus on other major injuries in multi-trauma patients. In such a high-stakes scenario, an automated method for Tile grading should ideally be transparent such that the symbolic information provided by the method follows the same logic a radiologist or orthopedic surgeon would use to determine the fracture grade. This paper introduces an automated yet interpretable pelvic trauma decision support system to assist radiologists in fracture detection and Tile grading. To achieve interpretability despite processing high-dimensional whole-body CT images, we design a neurosymbolic algorithm that operates similarly to human interpretation of CT scans. The algorithm first detects relevant pelvic fractures on CTs with high specificity using Faster-RCNN. To generate robust fracture detections and associated detection (un)certainties, we perform test-time augmentation of the CT scans to apply fracture detection several times in a self-ensembling approach. The fracture detections are interpreted using a structural causal model based on clinical best practices to infer an initial Tile grade. We apply a Bayesian causal model to recover likely co-occurring fractures that may have been rejected initially due to the highly specific operating point of the detector, resulting in an updated list of detected fractures and corresponding final Tile grade. Our method is transparent in that it provides fracture location and types, as well as information on important counterfactuals that would invalidate the system’s recommendation. Our approach achieves an AUC of 0.89/0.74 for translational and rotational instability,which is comparable to radiologist performance. Despite being designed for human-machine teaming, our approach does not compromise on performance compared to previous black-box methods.

## I. Introduction

Pelvic fractures are disruptions to the osteoligamnetous pelvic ring, located at the base of the spine [1] that require urgent medical treatment due to high angioembolization rates and high mortality [2], ranging from 4% to 15% [3] depending on fracture severity. Rapid assessment of pelvic fracture severity is important, because different fracture types and associated mechanisms of injury have varied levels of correlation with the need for urgent surgical or endovascular treatment to ensure optimal patient outcomes. Despite clear opportunities to provide actionable information, pelvic fracture severity grading remains a subjective bottleneck in patient triage. In contemporary practice, whole-body CT scans are routinely performed upon admission for trauma victims with suspected pelvic fractures [4], [5], which are then commonly graded with the unified AO Foundation/Orthopaedic Trauma Association (AO/OTA) system [6]. The CT scans are interpreted by radiologists with widely disparate levels of experience in a time-sensitive and high-stakes environment resulting in limited accuracy and inter-observer agreement [7]. An objective, automatic but interpretable approach that supports human-machine teaming is desired for AO/OTA Tile grading to overcome these issues. Interaction between artificial intelligence (AI) and radiologists could harmonize the accuracy of Tile grading among readers. Quick, automated but interpretable AI diagnosis ”can additionally serve as a pelvic fracture grading “second reader” that provides mental support and contributes to a faster and more objective interpretation process [8]. The interpretable AI in medical image analysis further extracts discriminative features transparent and understandable to radiologists to increase readers’ trust of AI predictions, thus addressing an important unmet clinical need [3]. A neurosymbolic system that closely mimics radiologist workflows, such as The AO/OTA Tile grading system, meets these requirements.

The AO/OTA Tile grading system separates injuries based on the stability of the pelvic ring (Type A- stable; B- rotationally unstable; and C- rotationally and vertically, or “globally” unstable). Higher severity grades correspond with the need for interventions including massive transfusion and angioembolization [9]. Different degrees of instability manifest with different types of abnormal bony relationships about the sacroiliac (SI) joint on CT scans. Complementary information is also gleaned from the anterior pelvic ring where unstable injuries result in pubic symphysis widening [6], [10]. Human readers explicitly search for evidence of rotational and translational instability separately, to arrive at a the Tile grade and this represents the most intuitive real-world approach to the problem. Therefore, an automatic and interpretable approach can predict AO/OTA Tile grading by following human search strategies to arrive at component instabilities (*rotational* or *translational*), as in [9], [10]. The relationship between pelvic fracture patterns and associated instability grades can be captured using an expert knowledge-based causal model approach that incorporates the fracture grading logic described by Marvin Tile [11], [12], [13].

In this paper, we present an automated yet interpretable algorithm for Tile AO/OTA grading from trauma CT scans. The system first identifies fracture types and locations via a deep object detection algorithm with augmentation-based self-ensembling inference for robust uncertainty quantification for fractures. Then the Tile grade is inferred from the types and combination of fractures with a Bayesian causal network. The framework provides a transparent inference pipeline that supplies fracture location and type, as well as information on counterfactuals, e.g., missed or misclassified fractures, that would invalidate the system’s recommendation. As such, the system is designed to decrease the workload of the radiologist or orthopedist by facilitating validation and refinement.

Based on the above, the contributions of our work can be summarized as follows:

- Like other automatic abnormality-finding algorithms, we train a multi-class detection network to detect fractures in whole-body CT scans. However, fracture detection is not the final goal of our proposed method. We further analyze the AO/OTA Tile grades which are directly relevant to clinical outcomes, interventions, and surgical planning.
- We follow the AO/OTA to derive a neuralsymbolic reasoning pipeline to learn and predict the Tile Grades with causal models.
- We further apply Bayesian causal models to retrieve low-confidence fractures which are erroneously rejected by the high-confidence operation point which is otherwise necessary to achieve high specificity.
- We introduce augmentation-based self-ensembling inference to calculate fracture confidence by voting statistics of slightly augmented CT scans. The voted confidence score is more robust and equitable compared to those directly calculated by the detection network.

Parts of this work were previously published as a conference proceeding [14]. This work considerbly expands on that initial approach adding several contributions: 1) We develop a 2.5-D algorithm to merge 2D fracture detections into 3D detections for further analysis, 2) We propose augmentation-based self-ensembling inference to robustly estimate the confidence scores of each detected pelvic fracture, 3) We include comprehensive results which are averaged across 5-fold cross-validation and provide a detailed analysis of algorithm hyperparameter behaviors.

## II. Related Work

### A. Fracture Classification and Detection

In the field of fracture detection and classification, studies have mostly focused on deep learning for plain radiography tasks [15], [16]. Literature on detection and classification of fractures using CT scans is limited [17], [18]. In classification tasks with radiographs, most of the studies [19], [20], [21] utilized state-of-the-art deep learning algorithms to achieve high metric performance. Some methods aim to standardize datasets to increase the ease of model training [22] or employ GAN-generated data [19] and multi-scale mechanism [20] to make models more generalizable. In detection tasks, most works use two-stage detectors, such as Faster RCNN [23] instead of one-stage detectors, such as YOLO [24] to achieve better performance [25], [26], [27]. Additionally, some researchers have tried to improve the Faster R-CNN using various approaches. [28] designed a guided anchoring method (GA), while [29] added a feature pyramid network to Faster R-CNN. [30] introduced a feature ambiguity mitigating operator for use in combination with different models. [31] engaged a triplet attention mechanism to obtain richer hairline fracture features. Chłąd et al. utilized vision transformer and cloud-based computation for cervical spine fracture detection [32]. Fracture detection in CT scans is quite challenging due to the lower in-plane resolution of CT slices and the complex curving 3D nature of the bony pelvis. Inoue et al. utilized Faster-RCNN-Inception-V2-COCO DCNN to automatically detect fractures in whole-body trauma CT [33]. Ukai et al. used DCNN-based YOLOv3 to detect fractures in images extracted from multiple orientations of 3D volumetric CT images [18]. Rahman et al. created synthesized pelvic x-ray images from CT scans [34]. Other reported methods utilized the clinical characteristics of bone structures to design fracture detection networks. Bilateral symmetry of pelvic structures are employed by Siamese networks with contrastive learning to explicitly detect fractures by bilateral asymmetric regions [35], [36]. Hsieh et al. designed a direction-aware deep learning algorithm to mark the exact femoral neck fracture location in radiographs [37]. We use a combination of object detection and bayesian causal modeling to provide interpretable first-order Tile AO/OTA grading.

### B. Features of Pelvic Ring Disruption

The AO/OTA has adopted the Tile classification scheme for grading mechanical instability and severity of pelvic fractures [38], in which the fracture grade has been shown to correlate with major arterial injury, need for surgical packing or catheter-based interventions, need for massive transfusion, and mortality [9], [39]. First order Tile grading employs the following scheme: **Grade A** - translationally (T) and rotationally (R) stable; **Grade B** - T stable and R unstable; and **Grade C** - both T and R i.e., “globally” unstable. Different degrees of instability manifest with different types of abnormal bony relationships on the CT scan, including pubic symphysis diastasis (PSD), divergent or parallel sacroischial (SI) joint diastasis, non-diastatic and diastatic sacral fractures, ischial spine avulsions (ISp), and innominate bone fractures involving the anterior and posterior pelvic ring [6], [10]. Fragility in elderly patients is not expressly included in Tile’s framework, but has come to be recognized as an important modifier of fracture severity [3]. Recently, deep learning approaches have been applied to automatically classify pelvic Tile grades. ResNet-50 was trained on a dataset of 373 trauma whole-body CTs collected from two busy level 1 trauma centers to predict tile grades [40]. A causal inference model was trained to reveal the relationships between fractures and predicted Tile grades [14].

### C. Explainable AI and Causality

Explainability attempts to reveal the working mechanisms of complex models. In [41], explainability is described not as a property of the machine learning model but as an affordance, i. e., a relationship between the algorithm and users. The AI system needs to convey the justification behind a predicted outcome as well as uncertainties [42] and robustness [43] of estimation. This necessitates a direct causal interaction between variables and the final prediction. Recent works in this direction explore the applicability of causality analysis for explainability of the medical diagnosis. [44] argue that how transparent and understandable model could be more closely related to the model size rather than specific model type. According to [45], careful interpretation of a diagram (in our case, for a widely used pelvic fracture scheme) gives insights about potential biases that are important to consider when designing experimental studies and drawing conclusions from statistical analysis. In [46], authors analyze whether diseases cause patients’ symptoms as a counterfactual inference task and derive counterfactual diagnostic algorithms, achieving expert clinical accuracy. An improved contrastive learning framework with adaptive anatomical contrast is proposed for semi-supervised medical segmentation where class distribution is also highly imbalanced [47]. [48] developed a new automated causal inference method (AutoCI) to evaluate the effectiveness of a vaccine against viral infection and applied this in two large-scale, practice-changing randomized controlled trials of patients with endometrial carcinoma conducted in the Netherlands. [49] presented an adversarial training strategy that allows a transformer-based discriminator to capture high-level semantically correlated contents and low-level anatomical features. Domain-specific spurious correlations are removed via causal intervention by independently resampling the appearances of potentially correlated objects [50]. Causal inference with plug-in clinical prior knowledge can also introduce transparency directly for various applications or domains [51], [52], [53].

## III. Method

**Fig. 1.**
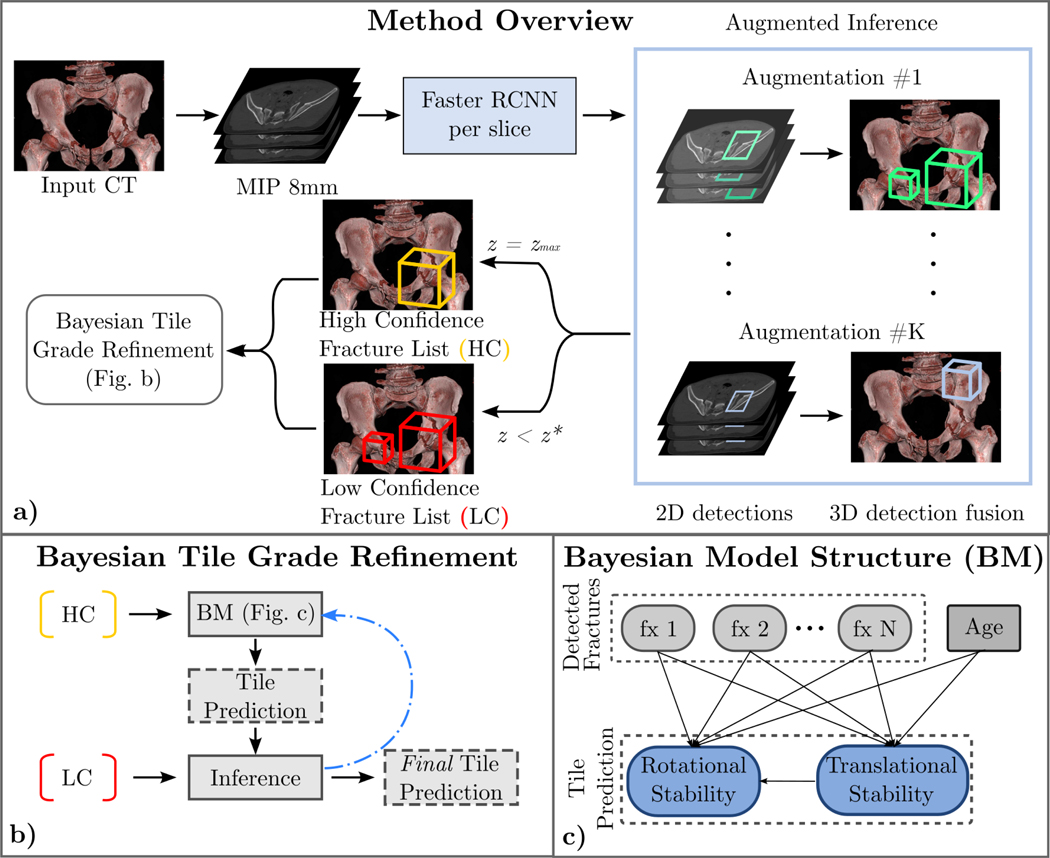
The pipeline of our system. a) Method overview. Faster RCNN is trained on CT scans with maximum intensity projectionss (MIPs) to detect key fracture classes that contribute to Tile grading. Then we apply augmentation-based self-ensembling inference to CT scans, where we create slightly augmented CT scans and detect fractures individually. We calculate fracture confidence for each detection by voting among all augmented CT scans. Finally, we extract high- and low-confidence fracture findings for BM analysis. b) Tile grade refinement algorithm using causal Bayesian model. Detailed description can be found in Algorithm 1. c) Structure of the BM: [fx 1 - fx N] are detected fracture types. Tile grade is represented by a combination of translational and rotational instability.

Given a whole-body CT scan, we aim to predict the AO/OTA Tile Grade of a given subject. The three Tile grades of the AO/OTA Tiles: **Grade A**, **Grade B**, and **Grade C** are determined by transitional (T) and rotational (R) instabilities. Different degrees of instability manifest with different types of abnormal bony relationships on the CT scan, as mentioned in Section II-B. Thus, we first apply Faster-RCNN [54] to detect the abnormal bony relationships, which are referred to as *fractures* or *joint space widening* in whole-body CT scans. The trained Faster RCNN detects each of the aforementioned features of pelvic ring disruption- for example, parallel SI joint widening, associated with vertical instability, is differentiated from anteriorly divergent SI joinnt widening, associated with rotational instability. Detected CT features have their own associated confidence scores. We fuse 2D fracture detections into 3D detections in each CT scan. However, the confidence scores of fractures calculated by Faster-RCNN are not the true confidence scores because they are directly affected by the loss weights and learning procedure. Furthermore, retrieval of false negative fractures from a CT scan is unlikely using single inference without uncertainty quantification. To alleviate these issues, we introduce an augmentation-based self-ensembling inference procedure, in which we augment the original CT scans multiple times and detect fractures in each augmented CT scan. Finally, voting among all augmented inferences is used to assess the confidence of each detected fracture. Details are shown in Figure 1a and Section III-A. Next, we obtain an initial Tile grade estimate from the Bayesian Model (BM) applied to the detected fractures with high confidence (see Figure 1b). The BM further suggests potentially missed (false negative) fractures that are rejected by a high confidence threshold, but accepted by a low confidence threshold. We add these potentially missed fractures by BM suggestions to the detected fracture list for final Tile grade prediction. This is further explained in Section III-B. Our method provides more accurate Tile Grading prediction by reducing the risks of missed fractures and false positives that occur with one-time fracture detection inference with non-robust network confidence scores; and also by identifying potential false negative findings that are erroneously rejected due to the clinical need for high precision.

### A. 2.5D Fracture Detection

The subset of pelvic fracture (fx)-related features that were determined a priori to be clinically meaningful for Tile grading by expert trauma radiologists using the Tile AO/OTA grading scheme includes: pubic symphysis diastasis (PSD); anteriorly divergent sacroiliac joint (SI) and parallel SI joint diastasis; non-diastatic and diastatic sacral fx; Ischial spine avulsion (ISp), and all remaining innominate bone fractures involving the anterior and posterior pelvic ring (henceforth “ring fx”). We use 2D Faster-RCNN to detect these clinically meaningful fractures with particular saliency for Tile AO/OTA grading. We apply maximum intensity projectionss (MIPs) in the axial axis to reduce the visual noise and artifacts in CT scan inputs. In order to make Faster-RCNN focus on the related pelvic fractures, the pelvis is manually cropped from the whole-body CT scan. After Faster-RCNN is trained, we first select all fractures detected by Faster-RCNN with network score > 0.5. We generate 3D detected features by fusing 2D detected fractures if the fracture types are the same and the 2D bounding boxes are overlapped and are located in adjacent slices. The network score of each 3D detected fracture is the maximum network score among all the corresponding fused 2D fractures.

Missed fractures (false negatives) in inference on CT scans have a direct impact on the Tile AO/OTA grading, because rotational and translational instability are highly related to the detected fractures and disruptions. There exist no effective approaches to retrieving the missed fractures in inference on the native CT scans. Additionally, there are occasional missed 2D fractures using Faster RCNN that may result in one true fracture splitting into multiple fused 3D detected fractures. We introduce augmentation-based self-ensembling inference to solve these issues. In addition to inference on original CT scans, we also apply detection model inference on CT scans that are slightly different from the original CT scans, which we call augmented CT scans. The augmentation operations are chosen so that the fractures are preserved in the augmented CT scans. Fracture detection inference on augmented CT scans can potentially detect new fractures that are missed in inference on the native CT scans. Furthermore, the augmented CT scans can be treated as “noisy” versions of the original CT scans, so that the fracture detections on each augmented CT scan can be used to calculate more robust confidence scores of the corresponding detected fractures. In detail, in addition to the original CT scan, we create K augmented versions of each CT scan. We apply the same fracture detection inference for all augmented CT scans. We fuse the 2D detections into 3D fracture detections. Finally, we fuse 3D fractures across all augmented CT scans if they are spatially overlapped and have the same fracture type. The spatial location of the fused 3D fractures fx is the smallest cuboid that contains all of the corresponding included 3D fractures. We use the **frequency** of the 3D fractures fx among all augmented CT scans as the final confidence score. The frequency is calculated by the number of augmented CT scans in which the included 3D fractures locate divided by the total number of augmented CT scans:

(1) $$P_{c}(fx)=\frac{{\sum_{k=1}^{K}1}_{\text{Fracture detected}}^{k}}{K}$$

where $P_{c}(fx)$ is the experimental confidence score of the fused 3D fracture fx; $1_{\text{Fracture detected}}^{k}$ means there contain fractures in the k-th augmented CT scan that are spatially included in the fused 3D fracture fx. Finally, the confidence of the existence of each fracture type in CT scans is equal to the maximum confidence score among all detected 3D fractures in the same fracture type.

**Fig. 2.**
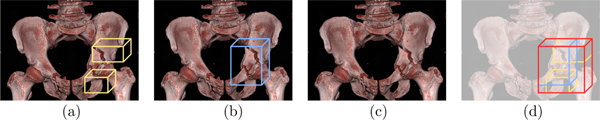
An example of 3D fracture fusion in augmentation-based self-ensembling inference. Figure (a), (b), (c) are 3 augmented CT scans. Boxes in (a) and (b) are 3D fractures fused from Faster RCNN results and are of the same fracture type. Red box in (d) is the final fused 3D fracture. Because fracture in (b) is intersected with both fractures in (a), they are fused and the red box in (d) is the final fused fracture. As the result, augmentation-based self-ensembling inference mitigates the 2D mis-detection (between the 2 yellow boxes in axial direction) in figure (a). The confidence score is the frequency score of fractures among all 3 augmented CT scans, which is 0.667.

Augmentation-based self-ensembling inference not only produces the frequency of 3D fractures instead of network scores which results in a more robust confidence score of detected fractures, but also mitigates the impact of the occasionally missed 2D fractures in Faster RCNN. For example, if we suppose one missed 2D fracture exists in the first augmented CT scan, which results in two 3D detected fractures of one true fracture, whereas all 2D fractures are detected in the second augmented CT scan. During the 3D fracture fusion among all augmented CT scans, the two 3D fractures in the first augmented CT scan are both fused with the one in the second augmented CT scan. In addition, because we calculate the frequency, the two 3D fractures in the first augmented CT scan are only counted once. A visual example is shown in Figure 2. Consequently, the occasionally missed 2D fractures in Faster RCNN do not impact the final frequency-based confidence scores of detected 3D fractures.

### B. Bayesian Learning

Given pelvic fracture detections, we need to predict the rotational and translational instability for AO/OTA Tile Grade. Using the close relationship between the selected fracture types and Tile Grade (mentioned in Section II-B), we train a causal model to predict Tile Grades with the detected fractures. Moreover, because of generalized bone loss with age, the bones of elderly people are fragile, which results in more frequent and complex pelvic fractures. We also add the age information of the patient as the input of the causal model. We created the causal graph illustrated in Figure 1c. All input nodes are formatted as boolean variables. We apply a threshold z to convert the frequency score of detected 3D fractures into boolean variables. The boolean variable of different fracture types is determined by the existence of the detected fracture of the corresponding fracture type by the pre-defined threshold z. The bool values of ages below 60 are 0, otherwise 1.

According to the clinical requirement to reduce the false positive rate of fracture detection, we apply a high operating point/threshold $z_{high}$ for the fused 3D fracture detections. However, some true fractures may be erroneously rejected due to the high confidence threshold $z_{high}$. To refine the Tile grade estimate based on these erroneously rejected fractures and simultaneously improve fracture detection, we intervene on the fused 3D fractures with high confidence $z_{high}$ to identify potential misses that would confirm or invalidate the current Tile estimate by the causal model. We check for the presence of these candidate fractures among all fused 3D fractures with confidence score $\in \left[z_{low},z_{high}\right]$, and if present, include them in our high-confidence findings. Finally, we run our BM on the updated list of fractures to obtain the binding estimate of AO/OTA Tile grade.

The proposed algorithm is formally described in Algorithm 1. An initial estimate of the Tile grade will be made by selecting the high-confidence fractures $FX_{high}$ of pelvic ring disruption with confidence threshold set to $z_{high}=z_{max}$ to range [0.8 − 1.0], so that the feature detection network will have high specificity. By computing the marginal likelihood probabilities for each possible Tile state, we get a list of potentially missed fracture labels that are more likely to co-occur ($p>z_{high}$) with the highly specific conditions found in the current CT scan. For every fracture in this candidate list, we check its presence in a list of lower-confidence fracture detections $FX_{low}$ that is generated using a lower threshold $z_{low}<z_{high}$. If such matching between the $FX_{low}$ and fractures suggested by BM is found and the predicted BM conditional probability is higher than threshold $z^{*}$, $FX_{high}$ will be updated by considering the additional features found in $FX_{low}$. Finally the predicted Tile grade will be updated based on this new fracture set.

While we consider our algorithm from a fully automated perspective in this manuscript, the refinement procedure based on a structural causal model is designed with immediate benefit for clinical workflows in mind. This is because, instead of setting a different threshold z for possibly missed fractures, our model could alert the attending radiologist for a review of the fractures. Similarly, the BM could calculate counterfactuals to the current prediction to identify possible misses that would change treatment recommendations. Because the object detection network supplies bounding boxes for each fracture finding, our method allows for effective visualization of all findings. This may contribute to an effective handover from an autonomous to a human-machine teaming operation.

## IV. Experiments and Results

### A. Datasets and Metrics

We obtained approval for the use of the dataset initially described in [40], which consists of 373 admission CT scans

**Algorithm 1:** Proposed Tile Refinement Algorithm Using a Bayesian Network Structural Causal Model (BM)

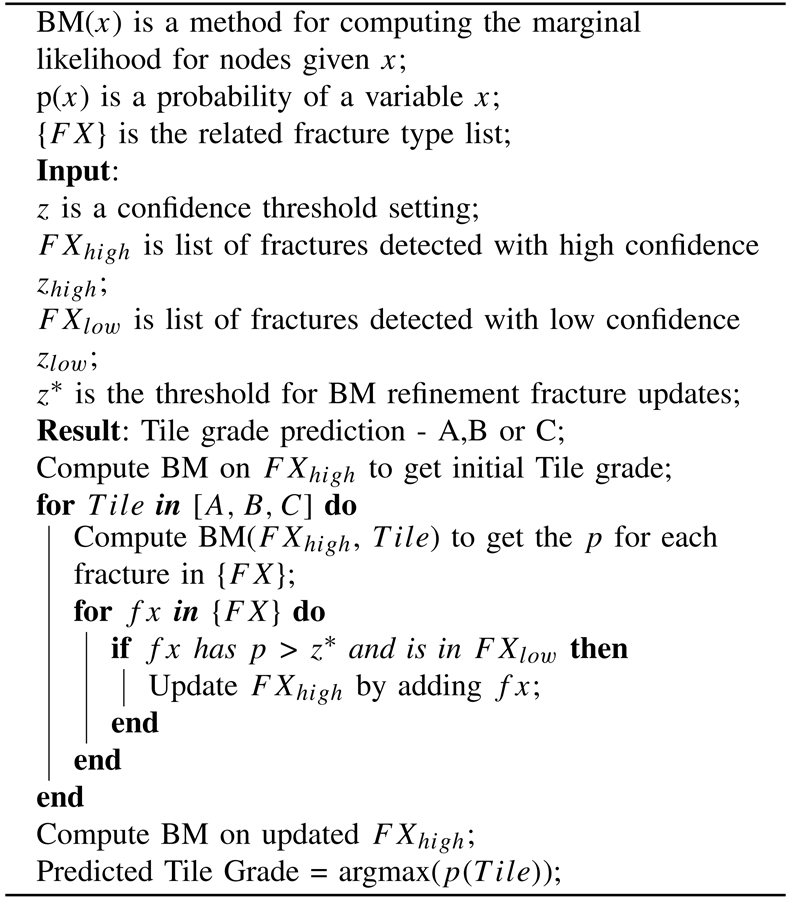

of adult patients from two level-I trauma centers with bleeding pelvic fractures and varying degrees of instability with IRB (Institutional Review Board)-approved waiver of consent. Imaging was performed with intravenous contrast in the arterial phase on 40-, 64-, and dual source 128-row CT scanners and reconstructed with 0.7 – 5 mm slice thickness. Patient-level annotation using the Tile AO/OTA grading system was performed by three trauma-subspecialized radiologists and the consensus vote was used as the gold standard for training and evaluation of the Bayesian causal model. The presence and location of fractures and fracture-related features such as pelvic ring disruption were performed on the 3D CTs by one radiologist with trauma expertise at the primary study site.

### B. Implementation Details

HU (Hounsfield unit) values of CT scans are first clipped to [−10, 1000] to highlight bone areas and rescaled into [0, 255] uint8 values. To preserve some volumetric information while reducing the complexity of the problem from 3D to 2D, MIP was used to generate 8 mm-thick slices along the axial plane of all CT scans. We found empirically that among various slice thicknesses, 8 mm thick MIPs yielded the best detection performance. Reference standard segmentation of pelvic fractures was propagated to these MIPs as bounding boxes with corresponding fracture labels. We manually cropped CT scans in the axial axis to only preserve slices within the pelvic region. On all MIPs containing labels, we train a Faster-RCNN using PyTorch [55] and Detectron2 [56] for 180,000 iterations with batch size 12 and a ResNXet-101_32 × 8d [57] backbone pre-trained on ImageNet to extract pelvic features. For evaluation, we used five-fold cross-validation where, in each fold, 80% of the data was used for training and 20% of the data was used for testing. Random flipping and random rotation were used for training data augmentation. During augmentation-based self-ensembling inference, we applied intensity shift $(I_{shift}\in [-2,-1,0,1,2]$ in uint8 image intensity) and contrast adjustment $(I_{contrast}\in [0.8,0.9,1.0,1.1,1.2])$ to create 25 augmented CT scans. We have manually checked that the visual feature of each fracture is preserved and almost unchanged. The BM is developed using the CausalNex [58] package. We fit probabilities of input variables in the causal model using Bayesian parameter estimation and learn conditional probability distributions (CPDs) w.r.t. Tile expert consensus from the same data split used during the fracture detection training. Code is available in https://github.com/hchen135/PelvicGrading.

### C. Experimental Results

#### 1) BM Refinement Performance:

To evaluate the Tile AO/OTA assessment, we split the Tile grade into component instabilities (*rotational* or *translational*), as in [9], [10]. Human readers explicitly search for evidence of rotational and translational instability separately to synthesize the Tile grade and this represents the most intuitive real-world approach to the problem. We present all scores as an average over the 5 folds in the cross-validation. We use the area under the receiver operating characteristic (ROC) curve (AUC) for classification performance and Kappa scores [59] for reader agreement measurements. In addition to our proposed algorithm, we also evaluate the performance using the network confidence score as the confidence score for detected fractures, instead of the ones calculated by the proposed augmentation-based self-ensembling inference. Detailed results are shown in Table I in the “2D Faster RCNN” section. Inter-observer reliability between automated predictions and expert consensus improved using the proposed refinement technique to a level that is close to the inter-rater variability of human observers. Since inter-observer reliability is only typically moderate to fair for many Tile grades [60], we hypothesize the lack of agreement is responsible for the imperfect performance of BM(GT) on expert-annotated fractures.

The Tile Grade performance is improved by higher confidence threshold of fractures. The refinement technique further boosts the Tile grade performance by retrieving true fractures that are erroneously rejected due to high precision clinical requirements. It achieves 0.89/0.74 AUC in translational and rotational instability, which is comparable to 77% interobserver fracture type agreement accuracy among radiologists [7]. It is also observed that the AUC and Kappa performance of all experiments with augmentation-based self-ensembling inference outperforms those without augmentation-based self-ensembling inference. The augmentation-based self-ensembling inference calculates the fracture confidence score in an experimental approach by slightly augmented “noisy” data, so that the confidence score is calculated more accurately than those by network confidence scores.

We also included results by replacing the 2D Faster RCNN with the 3D ones and skipped the 3D fracture fusion from 2D detections during the proposed augmentation-based self-ensembling inference, which is shown in Table I in the “3D Faster RCNN” section. Compared to 2D detection networks, 3D detection does not suffer slice-level discontinuity of fractures caused by 2D miss-detection, but it misses more rare, small, and non-displaced fractures. We observed similar performance in instability AUC between the 2D and 3D methods without augmentation-based self-ensembling inference (0.699 v.s. 0.695 in rotational instability and 0.848 v.s. 0.838 in translational instability). However, 2D detection network results with augmentation-based self-ensembling inference outperform the 3D ones (0.738 v.s. 0.714 in rotational instability and 0.890 v.s. 0.866 in translational instability). This indicates smaller performance improvement with augmentation-based self-ensembling inference to 3D detection network results because of non-retrievable missed small and non-displaced fractures. As a result, 2D detection methods outperform the 3D ones in the proposed pipeline for pelvic severity grading.

#### 2) Fracture Performance:

**Fig. 3.**
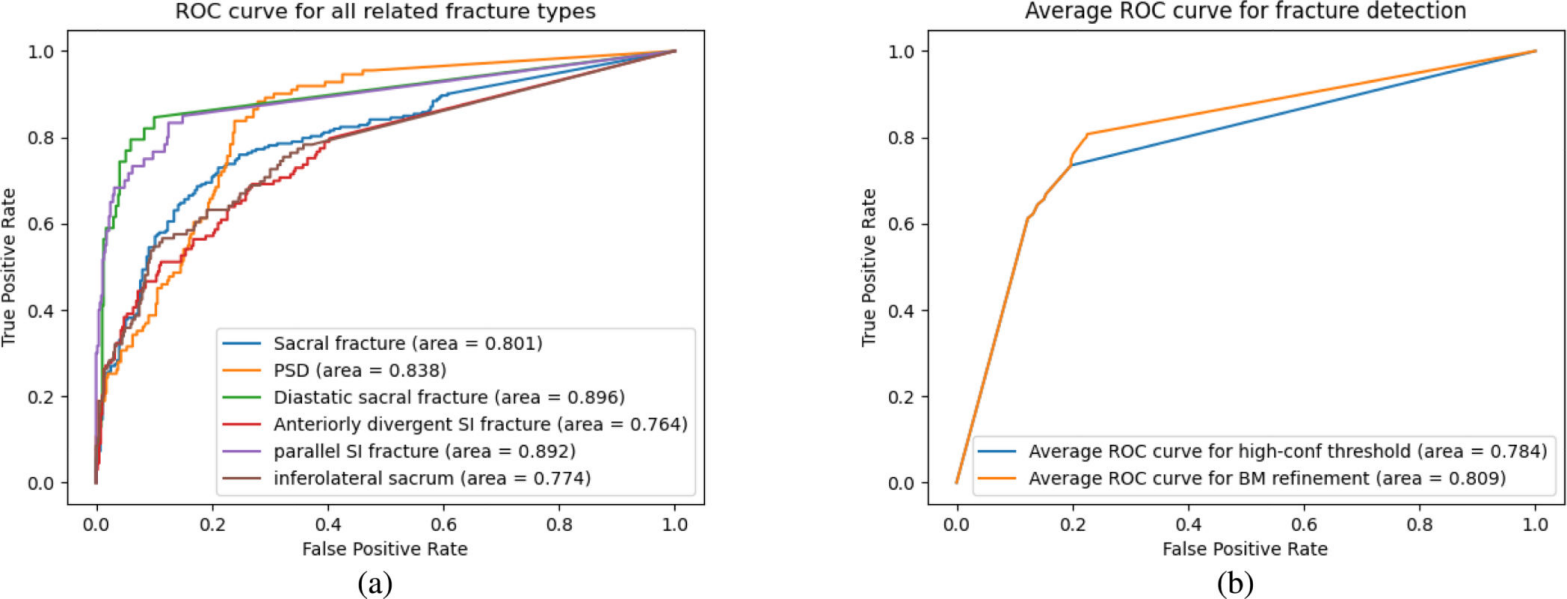
(a) ROC curves for every fracture type using Faster-RCNN and augmentation-based self-ensembling inferencee at z=max. (b) Average ROC curve of Faster-RCNN-based fracture detection with and without BM-based refinement for z=max. Results shown with 5-fold cross-validation.

**Fig. 4.**
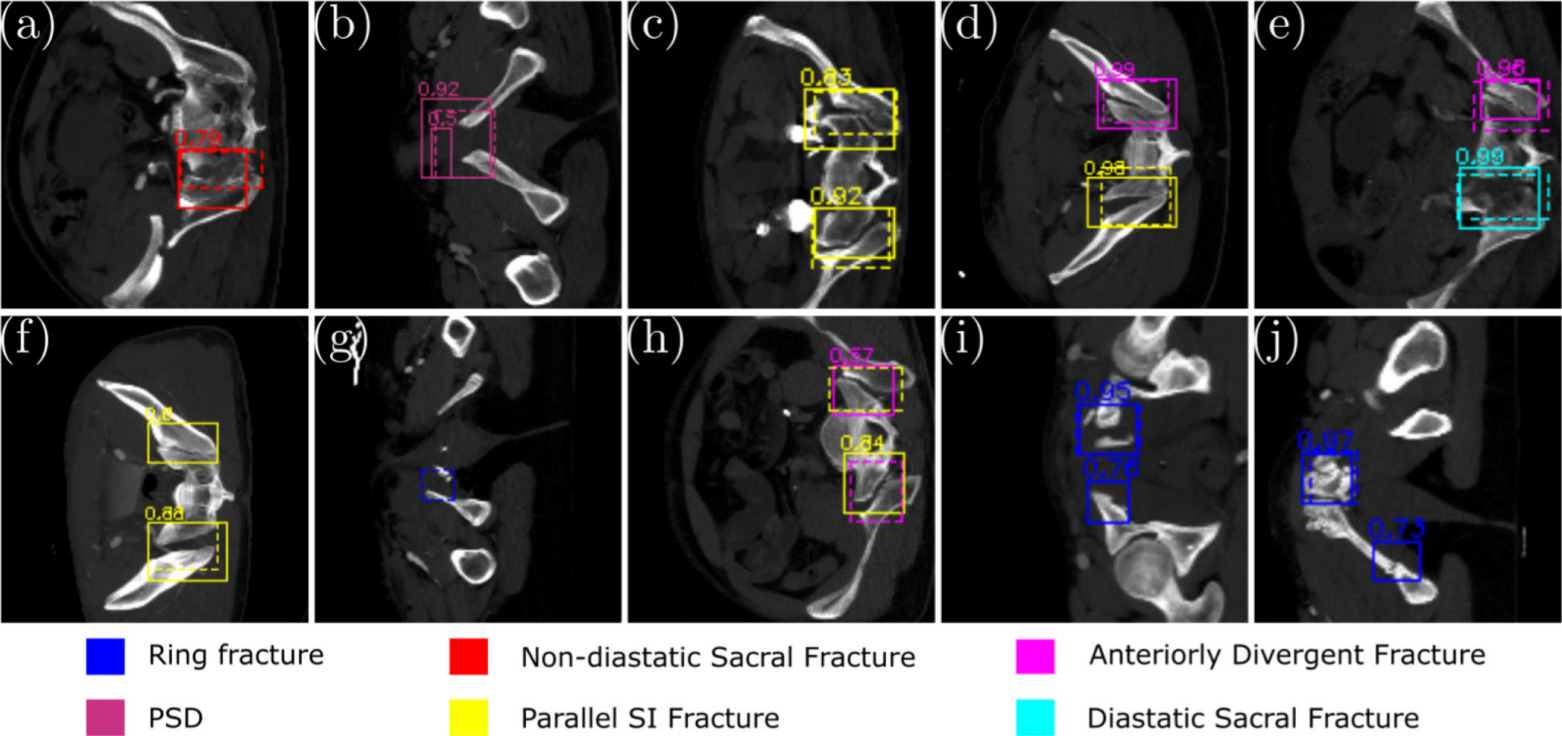
Visualization of 2D fracture detection results. Solid rectangles are the detected fractures. Dashed rectangles are ground truth. Fractures in the first row (a, b, c, d, e) are correctly detected. Fractures in the second row (f, g, h, i, j) are not corrected detected.

We evaluated Faster-RCNN for each fracture type separately (Figure 3a). The AUC of different pelvic fracture types all reached more than 0.75. Some fractures like diastatic sacral fracture reached an AUC of 0.896. Consequently, the high accuracy of 2D detection pelvic fractures by Faster RCNN supports the following analysis with Bayesian models. Figure 4 also shows the visualization of 2D fracture detections. Figure 4 (a, b, c, d, e) shows fractures that are correctly detected. Figure 4 (f, g, h, i, j) shows different missed fractures. Many false positives occur in the symmetric fracture regions (Figure 4 (f, i), indicating the detection network is comparing bilateral symmetric areas for final predictions. Other miss-detections happen in the joint between the sacral promontory and wing of the ilium such as Figure 4 (h), because the difference among parallel SI fractures, anteriorly divergent fractures, and diastatic sacral fractures are highly related to the joint separation configurations, which may have small differences in Tile B and C edge cases. There also exist fractures that are not completely labeled, such as Figure 4 (j). By the analysis of fracture types in Bayesian models, we found that the fracture with the lowest confidence by the object detector (Anteriorly Divergent SI) is often responsible for the Tile prediction update. Even though Anteriorly Divergent SI fractures were initially left out by a high confidence threshold, anteriorly divergent SI fractures were included after the BM refinement. In a situation where a binder (a device used to stabilize suspected pelvic fractures in patients with traumatic injury) is present, the subset of visible fractures will appear less severe, even though the presence of Anteriorly Divergent SI indicates a major high energy blunt trauma event. We then evaluate the performance of Faster-RCNN for fracture detection with and without BM-based refinement(Figure 3b) and show that the proposed scheme not only improves Tile grading but also positively affects fracture detection when the initial network operates at high specificity.

#### 3) Age Impact in Bayesian Model Performance:

We performed experiments without age variable in the BM as a comparison with the proposed method. The age information is eliminated in the input data as well as in the structure of the BM. The refined BM achieved 0.871/0.752 AUC in translational and rotational instability. The AUC for rotational instability is higher when age is not considered, while the AUC for translation instability is lower. This also fits the real clinical practices for pelvic fracture severity grading. If an elderly person has pubic symphysis diastasis, clinicians additionally examine sacral fractures for diastasis, according to the Tile AO/OTA system, to determine whether the patient has translational instability or not, which results in a low translational instability AUC in the Bayesian model without age input.

#### 4) Confidence Threshold Behavior:

**Fig. 5.**
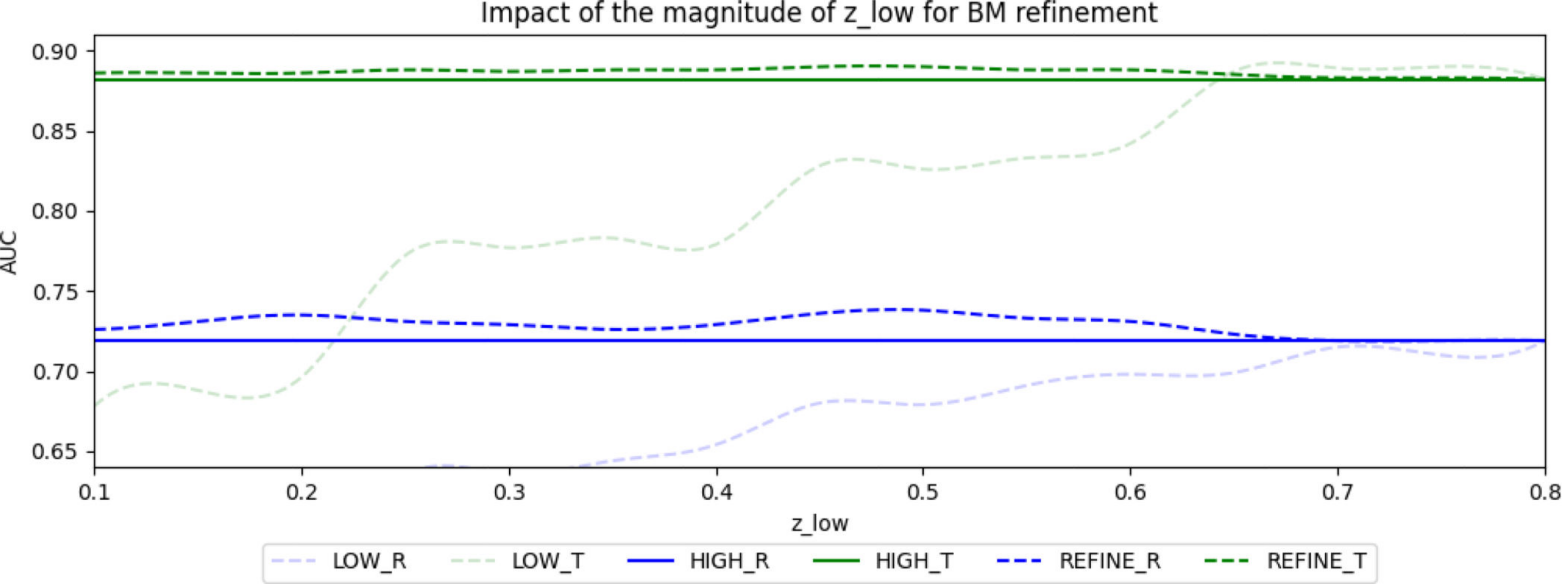
Impact of the magnitude of $z_{low}$ of $FX_{low}$ on the AUC of Tile prediction for BM refinement. We fixed $z_{high}=0.8$ and $z^{*}=0.5$. The plot shows the behaviors of BM model prediction for subjects with fractures accepted by low-threshold (**LOW**), high-threshold (**HIGH**), and updated BM refinement (**REFINE**) for rotation (**R**) and translation (**T**). The proposed method outperforms the BM without refinement in all values, indicating that with proper $z_{high}$ and $z^{*}$, BM with refinement guarantees to boost the performance for rotation and translation instability prediction.

There exist 2 confidence thresholds/hyperparameters in our algorithm: the low confidence threshold of candidate fractures $z_{low}$, and the fracture list refinement threshold for BM conditional probability $z^{*}$. We evaluate the impact of the 2 thresholds separately by comparing the performance of BM with/without refinement ($FX_{high}$ v.s. BM refinement) on predicting Rotational (R)/Translational (T) instabilities. $FX_{low}$ is visualized as a reference for comparison. We first assess the impact of low confidence fractures with $z=z_{low}$ to the performance boost in BM refinement, which is shown in Figure 5. We set $z_{high}=0.8$ and $z^{*}=0.5$ for this experiment. The AUC performance of BM refinement outperforms the ones without BM refinement in all different $z_{low}$ thresholds to determine the fracture candidates, indicating that with carefully selected $z_{high}$ and $z^{*}$, $z_{low}$ does not have a significant impact on the boosted performance of BM refinement.

**Fig. 6.**
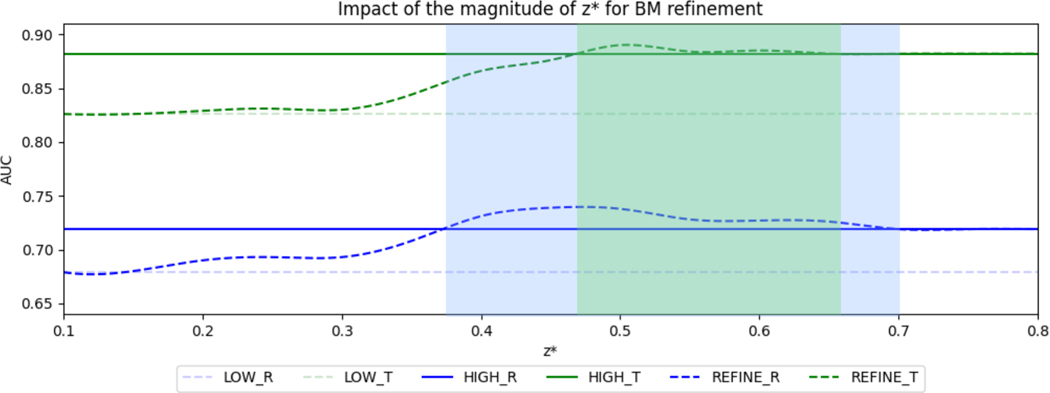
Impact of the magnitude of fracture update confidence $z^{*}$ on the AUC of Tile prediction for BM refinement. We fixed $z_{\text{high}}=0.8$ and $z_{low}=0.5$. The plot shows the behaviors of BM model prediction for subjects with fractures accepted by low-threshold (**LOW**), high-threshold (**HIGH**), and updated BM refinement (**REFINE**) for rotation (**R**) and translation (**T**). The blue and green opaque areas show the intervals where BM refinement boosts the performance for rotation and translation predictions, respectively. The proposed method outperforms the BM without refinement when $z^{*}\in [0.45,0.65]$, and therefore is less dependent on the initial parameter selection.

We also evaluate the impact of the fracture list update threshold $z^{*}$ on BM refinement performance, which is shown in Figure 6. We set $z_{high}=0.8$ and $z_{low}=0.5$ for this experiment. We observe that both rotation (R) and translation (T) are improved with BM refinement when $z^{*}\in [0.45,0.65]$, which indicates that the proposed refinement method improves upon direct inference on a less specific detection result without being overly dependent on the hyperparameter for fracture list updates.

## V. Discussion

### A. Comparison With Previous Work

#### 1) The Selection of 2D Faster RCNN as the Detection Network:

Both 2D and 3D detection networks would be applicable in pelvic fracture detection scenarios because the detection network aims to identify the existence of different fracture types in the CT scans, which is the input of Bayesian inference. Although 3D network does not suffer mis-fusion of 3D fractures caused by 2D miss-detection, we choose the 2.5D detection method instead of the 3D ones because 3D detection network fails to detect many small and non-displaced fractures, which are also less likely to retrieve by bayesian modeling, resulting in worse performance in final severity grading.

There also exist many 2D detection networks, but we choose the anchor-based Faster RCNN instead of prototype-map based YOLO [61]. The detection network aims to detect fractures and the existence of different fracture types is further used as input in Bayesian models. We choose anchor-based Faster-RCNN because the anchor algorithm is more straightforward for clinicians. In contrast, prototype-map-based methods like YOLO extract bounding boxes based on linear combinations of probability maps, which requires more math and statistical background to understand. All in all, we choose Faster-RCNN methods, but we agree that YOLO should also work on this task.

#### 2) The Selection of Bayesian Model as the Final Classification Method:

Causal inference is highly desired in computer-aided diagnosis (CAD) for clinical practice. Causal inference following clinical guidelines used by clinical experts makes results to be more interpretable and reliable, because such CAD inference follows the same decision-making process as clinical experts. The proposed Bayesian inference to retrieve pelvic fractures following AO/OTA grading is one application of causal inference application for clinical AI. In clinical practice, radiologists find fractures and use the relationship between fracture existence, age information, and fracture severity grading to make clinical diagnoses. Expert knowledge-based Bayesian models perform classification using the same logic and symbolic information that a radiologist or clinician would use to come to a given diagnosis, and therefore alligns well with clinical practice. In contrast, classic ensembling methods like Random Forest can hardly express, in an interpretable fashion, the relationship between input variables and the output fracture severity grading from the thousands of trees trained in the random forest. Another major justification for using Bayesian models for pelvic severity grading is that Bayesian models can retrieve missed fractures from low threshold $z_{low}$ by moving complementary AO/OTA Tile Grades into inputs to predict initially undetected fractures with high threshold $z_{high}$. Other classic methods, such as logistic regression do not have this retrieval ability. Such causal inference methods may also be applied to other clinical tasks such as employing clinical taxonomy to classify abnormalities in chest X-rays or utilizing the AAST criterion for splenic injury grading.

#### 3) Comparison Between Augmentation-Based Self-Ensembling Inference and Training Data Augmentation:

Training data augmentation increases input variety and results in more generalizability of network models. As a result, we also have applied augmentation of CT scans as inputs during training of the detection network. However, we observe that some fractures are correctly detected but received comparatively low network scores. These detected fractures are commonly small fractures or non-displaced fractures, whose visual pattern is not as obvious as other large and displaced fractures. We believe that the low network scores of small or non-displaced fractures stem from the nature of kernel and pooling-based learning, where small features are mostly filtered out because of the network structures. The low network scores of such fractures can be resolved by self-ensembling inference. Self-ensembling inference calculates the frequency of fractures in all augmented CT scans instead of using network scores, which greatly reduces the impact of network structures on the final confidence scores.

### B. Innovations and Limitations

#### 1) The Ability of Self-Ensembling Inference to Generate Robust Uncertainty Scores and Retrieve 3D Missed Fractures in Detection Network:

There is interest in associating uncertainty to deep learning outputs to reduce the impact of uncertainties [62] and increase prediction robustness [43] during the decision making process. There are several different methods to quantify uncertainty, such as bayesian methods: Monte Carlo test-time drop out [63], Bayes by BackProp [64], variational autoencoder [65]; and ensemble methods: test-time ensembling augmentation [63]. To our best knowledge, the proposed efficacious test-time augmentation represents a novel approach. It can be applied in both 2.5D and 3D detection results. In 2.5D detection, it also resolved 3D fracture fusion error caused by occasionally miss-detected 2D fractures, because augmentation-based self-ensembling inference also fused intersected 3D fractures among augmented CT scans. Augmentation-based self-ensembling inference can also be applied in multiple GPUs simultaneously so that the inference time is not significantly increased compared to classical inference. In this way, 2D detection methods outperform 3D methods with augmentation-based self-ensembling inference in processing speed, a hard requirement in automatic pelvic fracture detection.

The issue of missed fracture detections comes from low confidence scores or complete mis-detection of true fractures. There is no way to retrieve true fractures that are completely missed, while test data augmentation with self-ensembling inference aims to rescue only those true fractures with low but non-zero network confidence scores. Low network scores mainly stem from two factors: 1) The visual pattern is an outlier from the training set; 2) The pattern is subtle. There are lots of fractures that are non-displaced and fall into the latter group. Low network scores of such fractures do not mean the network does not detect them, but may rather be due to the nature of the network’s kernel and pooling-based decision making, which eliminates small visual patterns in feature encoding. In contrast, in test data augmentation with self-ensembling, we instead calculate the frequency of 3D fractures existing in all augmented CT scans as a representation of the confidence of 3D fracture detections. In this way, subtle non-displaced fractures with under-estimated network scores can still be retrieved by the self-ensembling inference.

#### 2) Augmentation-Based Self-Ensembling Inference Mitigates the Impact of 2D Mis-Detection in Faster RCNN:

2D fractures missed by Faster RCNN may result in disconnection of 2D slices within a true 3D fracture, leading to the generation of multiple separate 3D fused fractures for a single true fracture. To overcome this issue, a two-step fracture fusion strategy is proposed within the framework of augmentation-based self-ensembling inference. The first step involves the fusion of 2D network detections into 3D counterparts if they satisfy specific criteria: intersection in the axial plane, belonging to the same fracture type, and occurring in the same or consecutive slices. This fusion process aims to address instances where 2D fractures intersect but are separately detected within the same slice. The second step entails the fusion of 3D fractures across all augmented CT scans if they intersect in 3D space and belong to the same fracture type. This step serves to conform any true fractures that were separated during the initial fusion process due to mis-detections by the 2D Faster R-CNN. It is noted that the mised 2D fractures are likely to be captured in at least one augmented CT scan. As the result, such fracture separation is eliminated with the help of the second step fusion. There is thus low probability of a separated 3D fracture remaining disconnected from other fractures of the same type across all augmented CT scans. Consequently, these separated 3D fractures receive low confidence scores based on robust confidence calculations derived from frequency scores among intersected fractures in augmented CT scans. As a result, such fractures are not considered in Bayesian refinement, as their confidence scores fall below the specified low threshold of 0.5.

### C. Clinical Importance

#### 1) The Proposed Method has High Potential to be Interpretable to Radiologists:

The proposed method uses clinical guidelines in combination with causal principles to devise neurosymbolic AI for pelvic severity grading, mimicking the daily clinical practice of radiologists, by identifying the existence of different fracture types and considering explicitly the relationship between the fracture type and other clinical variables such as age in the final grade decision making. The proposed method also takes into account the potential risks of missed fracture using a process of retrieval that leverages the relationship between fracture types and Bayesian Model severity grades. A user interface can provide supportive causal information from the proposed inference and allow radiologists or surgeons to quickly verify or reject results by inspecting the symbolic information. Such a user interface with causal information following clinical guidelines may therefore provide useful, intuitive, and high-trust pelvic fracture severity grading information known to correlate with a variety of patient outcomes.

In future work, we aim to design a user interface in collaboration with radiologists. We can first develop user interface (UI)prototypes and then perform formative user studies with public datasets and refine the prototypes with iterative expert end-user feedback, to ensure a high degree of trust, mental support, low frustration, and high likelihood of future use. The UI can be integrated into a hospital picture archival and communication system (PACS) and deployed on hospital workstations for pre-clinical shadow testing. We may also utilize data-protective approaches, such as federated learning to iteratively update models according to the injury prevalence encountered in trauma centers with, for example, varying volumes, catchment areas, and rural versus urban settings.

Another future avenue may involve exploring the effectiveness of our augmentation-based self-ensembling inference and causal bayes models for additional clinical applications that require transparent inherently interpretable grading, such as for solid organ severity in the trauma domain, or for cancer detection and staging in oncologic imaging.

## VI. Conclusion

In this work, by leveraging the respective strengths of deep learning for feature detection and a BM for causal inference, we achieve automatic and interpretable Tile AO/OTA first order grading of pelvic fractures with pelvic CT. Using the proposed pipeline, Tile grade prediction had an AUC of 88.9% and 73.8% for translational and rotational instability, respectively, comparable to a previous black-box method [40] but with added interpretability and potential for human-machine teaming. We also proposed augmentation-based self-ensembling inference to estimate confidence scores of fracture findings, which is more robust and equitable because it is calculated by fractures detected in similar augmented CT scans.

The described methodology can benefit from interaction with experienced radiologists in place of the autonomous refinement, to further increase the inter-observer agreement. One limitation to be addressed in the future, either algorithmically or through interaction, are false-positive detections, i.e., false positives that are detected with high confidence and then propagated unchanged through the refinement step. Because our BM-based refinement strategy cannot currently reject high-confidence fractures, these findings would irrevocably bias the Tile grade estimation. Another line of future work will include investigations on the effectiveness of the proposed system when working with attending trauma radiologists in a collaborative setting to see if the system can indeed decrease the time to triage. We anticipate that the core ideas of the presented framework may find application across various diagnostic image interpretation tasks. We understand our methods as a step towards machine-human teaming for quick and accurate pelvic fracture severity scoring using the Tile AO/OTA system to standardize and accelerate triage, and ultimately, reduce mortality rates.

## Figures and Tables

**TABLE I:** Average AUC and Kappa Scores (Higher the Better) for Rotational (R) and Translational (T) Instabilities With 2D and 3D Fracture Detection Models. All Columns Are Compared to the Patient-Level Expert Consensus Tile Grade by Three Radiologists. We Evaluate Two Algorithms: **BM** Represents Bayesian Model (BM) With Network Confidence Scores. **BM_AugInf** Represents BM With Confidence Scores Calculated by Augmentation-Based Self-Ensembling Inference. We Also Compare Algorithms With Different Fracture Detection Results: ***GT*** Uses Annotated Fractures, ${\text{FX}}_{\text{LOW}}$ Uses Automatically Detected Fractures When Lower Confidence Fractures (With z=0.5) Are Immediately Included In Tile Grade Inference; ${\text{FX}}_{\text{HIGH}}$ Represents BM Prediction on Automatically Detected Fractures With High Confidence (z=max); ***refinement*** Represents Predictions After the Proposed Refinement Pipeline. $z^{*}=0.5$ for All Experiments With Refinement Fracture Updates. Results Shown With 5-Fold Cross-Validation

Metrics	AUC (R)	AUC (T)	Kappa (R)	Kappa (T)
BM(GT)	0.860 ± 0.007	0.907 ± 0.003	0.533 ± 0.023	0.489 ± 0.031
2D Faster RCNN				
BM $\left(FX_{low}\right)$	0.647 ± 0.003	0.791 ± 0.005	0.080 ± 0.021	0.333 ± 0.018
BM $\left(FX_{high}\right)$	0.685 ± 0.004	0.851 ± 0.004	0.132 ± 0.012	0.407 ± 0.012
BM refinement	0.699 ± 0.004	0.848 ± 0.006	0.145 ± 0.011	0.407 ± 0.013
BM_AugInf $\left(FX_{low}\right)$	0.679 ± 0.006	0.826 ± 0.003	0.143 ± 0.024	0.384 ± 0.030
BM_AugInf $\left(FX_{high}\right)$	0.719 ± 0.003	0.882 ± 0.005	0.248 ± 0.022	0.503 ± 0.013
BM_AugInf refinement noAge	**0.752 ± 0.007**	0.871 ± 0.007	**0.349 ± 0.015**	0.509 ± 0.026
**BM_Auglnf refinement**	0.738 ± 0.008	**0.890 ± 0.007**	0.268 ± 0.013	**0.535 ± 0.029**
3D Faster RCNN				
BM $\left(FX_{low}\right)$	0.687 ± 0.004	0.818 ± 0.003	0.170 ± 0.008	0.413 ± 0.004
BM $\left(FX_{high}\right)$	0.689 ± 0.006	0.835 ± 0.003	0.199 ± 0.009	0.485 ± 0.006
BM refinement	0.695 ± 0.005	0.836 ± 0.003	0.204 ± 0.010	0.485 ± 0.006
BM_AugInf $\left(FX_{low}\right)$	0.701 ± 0.007	0.844 ± 0.002	0.255 ± 0.009	0.431 ± 0.007
BM_AugInf $\left(FX_{high}\right)$	0.709 ± 0.008	0.850 ± 0.002	0.281 ±0.011	0.436 ± 0.004
BM_AugInf refinement noAge	0.734 ± 0.008	0.853 ± 0.003	0.304 ± 0.008	0.443 ± 0.009
**BM_AugInf refinement**	0.714 ± 0.008	0.866 ± 0.002	0.285 ± 0.012	0.485 ± 0.006
